# Interobserver Reliability of the Paris Classification for Superficial Gastrointestinal Tract Neoplasms: A Systematic Review

**DOI:** 10.1093/jcag/gwad039

**Published:** 2023-10-10

**Authors:** Sarang Gupta, Sam Seleq, Nikko Gimpaya, Rishad Khan, Michael A Scaffidi, Rishi Bansal, Samir C Grover

**Affiliations:** Department of Medicine, University of Toronto, Toronto, ON M5B 1W8, Canada; Department of Medicine, University of Toronto, Toronto, ON M5B 1W8, Canada; Division of Gastroenterology, St. Michael’s Hospital, Toronto, ON M5B 1W8, Canada; Division of Gastroenterology, St. Michael’s Hospital, Toronto, ON M5B 1W8, Canada; Li Ka Shing Knowledge Institute, University of Toronto, Toronto, ON M5B 1W8, Canada; Department of Medicine, University of Toronto, Toronto, ON M5B 1W8, Canada; Division of Gastroenterology, St. Michael’s Hospital, Toronto, ON M5B 1W8, Canada; Division of Gastroenterology, St. Michael’s Hospital, Toronto, ON M5B 1W8, Canada; Department of Medicine, University of Toronto, Toronto, ON M5B 1W8, Canada; Division of Gastroenterology, St. Michael’s Hospital, Toronto, ON M5B 1W8, Canada; Li Ka Shing Knowledge Institute, University of Toronto, Toronto, ON M5B 1W8, Canada; Scarborough Health Network Research Institute, University of Toronto, Toronto, ON M1P 2V5, Canada

**Keywords:** gastrointestinal neoplasms, colorectal polyps, Paris classification, interobserver reliability

## Abstract

**Background and study aims:**

The Paris classification characterizes the morphology of superficial gastrointestinal tract neoplasms. This system has been shown to predict the risk of submucosal invasion in certain subtypes of lesions. There is limited data that assesses its agreement amongst endoscopists. We performed a systematic review to summarize the available literature on the interobserver reliability (IOR) of the Paris classification.

**Methods:**

We conducted a search through December 2020 for studies reporting IOR of the Paris classification. Studies were included if they quantitatively evaluated the IOR of the Paris classification with at least five participating endoscopists. Two authors independently screened studies and abstracted data using an a priori-designed data collection form. Evaluation of study quality and risk of bias was performed using an adapted version of the Guidelines for Reporting Reliability and Agreement Studies.

**Results:**

Of the 1,541 studies retrieved, 5 were included in the review. All studies were observational cohort studies published between 2014 and 2020. The IOR of the Paris classification was moderate amongst all four studies evaluating colorectal neoplasms (range, *κ* = 0.42 to *κ* = 0.54) and substantial in one study that evaluated gastric neoplasms (κw = 0.65). An educational intervention was conducted by three studies with variable methodology and no significant change in IOR.

**Conclusions:**

IOR of the Paris classification is moderate for superficial colonic neoplasms. Further study is needed to determine the reliability of this system for superficial gastric lesions. Standardized training programs are required to investigate the impact of educational intervention on the Paris classification amongst endoscopists.

## Graphical Abstract

**Graphical Abstract:**
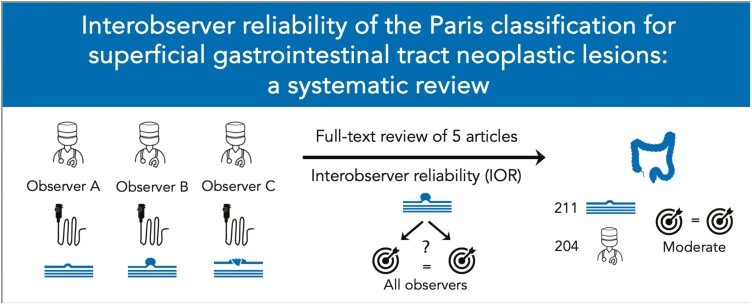

## Introduction

With an estimated incidence of 3.6 million cases, malignancies of the luminal gastrointestinal tract comprised almost one-fifth of all cancers in 2020.^1^ Early precancerous forms of these neoplasms are those that are confined only to the superficial layers of the gastrointestinal tract. Careful examination with various endoscopic tools and morphological classification systems can allow endoscopists to identify precancerous polyps with features of submucosal and nodal invasive disease.^2–4^

Developed in 2002 by a group of Western and Japanese physicians, the Paris Classification of Neoplastic Lesions of the gastrointestinal tract is one such prevalent morphological classification system that has demonstrated, that its, different subtypes are predictive factors for the risk of submucosal invasion.^5,6^ For example, while less frequently encountered, especially in Western studies, colonic lesions that are excavated or depressed are generally known to harbour a higher risk of invasion.^7^ As recommended by the US Multi-Society Task Force on Colorectal Cancer, the Paris classification should be used as part of the initial assessment of colorectal polyps to assist in guiding endoscopic or surgical management.^8^

There has, however, been some data to suggest only moderate interobserver reliability (IOR) of this classification system among users as well as considerable variation in the classification of flat (non-polypoid) lesions among expert endoscopists.^9^ Moreover, despite its widespread use in clinical decision-making and comparative endoscopic research, the data surrounding its reproducibility amongst endoscopists is limited. As such, we performed a systematic review to investigate the IOR of the Paris classification system.

## Materials and methods

We conducted a systematic review that was reported as per the Preferred Reporting Items for Systematic Reviews and Meta-analyses (PRISMA) recommendations (Supplementary Table 1).^10^ The protocol was registered on PROSPERO (CRD42021247175).

### Search strategy

We searched the online biomedical databases MEDLINE, EMBASE (Excerpta Medica Database), Scopus, CINAHL Complete, and Cochrane Library with the assistance of an information scientist. Searches were executed on December 31, 2020. An example of a search query conducted for MEDLINE is demonstrated in Supplementary Table 2.

### Study selection

After duplicate records were removed, two reviewers independently screened all abstracts to identify any for full-text review. Any inter-reviewer discrepancies were resolved through discussion and consensus.

### Eligibility criteria

We included a study if it reported a quantitative value on the IOR of the Paris classification, had a minimum of five participating endoscopists, was published in English, and was available in full-text format. We excluded studies for any of the following reasons: published as abstracts, conference papers, commentaries, and case reports; no reported quantitative value for the IOR; and/or evaluation of the IOR for other morphological features (e.g., size, mucosal surface pattern) in isolation. If relevant data were not available in the primary manuscript, we contacted the corresponding authors in an attempt to increase the sample size.

### Data extraction and quality assessment

A standardized data abstraction form was created *a priori* to extract prespecified variables from each included study. Data extraction was performed independently and blinded by two reviewers, wherein any inter-reviewer discrepancies were resolved through discussion and consensus.

Two reviewers independently assessed study quality and risk of bias using a tool adapted from the Guidelines for Reporting Reliability and Agreement Studies.^11^ We defined an article as “high quality” for a score of ≥11, “moderate quality” for a score of between 8 and 10, and “low quality” for a score of ≤7. Inter-reviewer discrepancies were resolved through discussion and consensus.

### Outcome measures

The primary outcome was the IOR of the Paris classification for superficial gastrointestinal tract neoplastic lesions. Secondary outcomes evaluated the effects of an educational intervention on the IOR.

Agreement analysis is described based on the number of raters and possible responses per rater. When there are two raters involved, Cohen’s kappa (*κ*),^12^ Scott’s Pi,^13^ or Gwet’s AC1 coefficient^14^ can be used. Fleiss kappa (*κ*)^15^ is used when there are three or more raters with the possibility of three or more responses per rater. *κ* can be weighted to account for the degree of difference between observers (*κw*). Proportion of agreement or pairwise agreement (PA) can also be used to describe inter-rater reliability; however, unlike kappa statistics, these do not account for the possibility of agreement occurring by chance.^11^

Interpretation of *κ* statistics followed standard values, wherein *κ* = 0.0–0.20 indicates “slight agreement”, *κ* = 0.21–0.40 indicates “fair agreement”, *κ* = 0.41–0.60 indicates “moderate agreement”, κ=0.61–0.80 indicates “substantial agreement”, and *κ* = 0.81–1.0 indicates “almost perfect agreement” as per Landis and Koch.^16^

## Results

### Study selection

**Figure 1.**
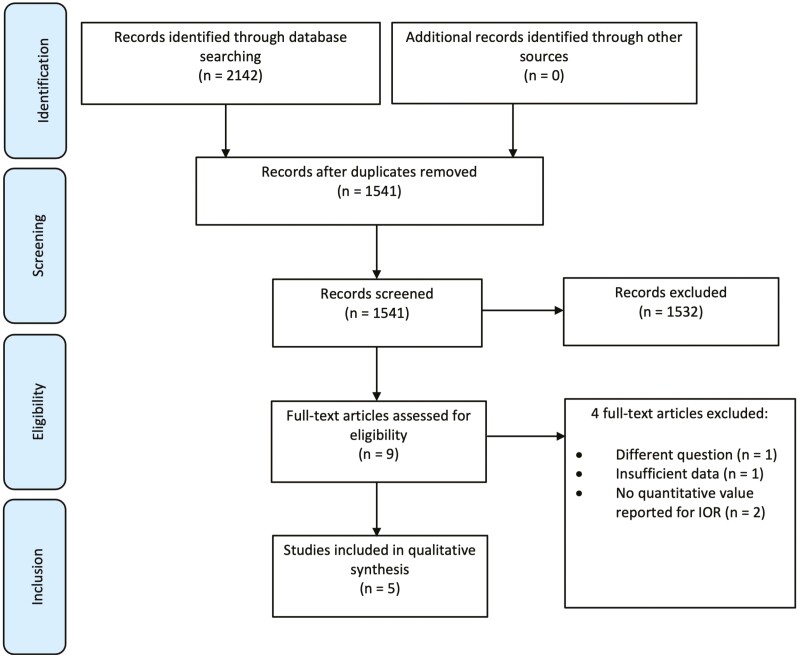
PRISMA flow diagram.^17^

The study flow is outlined in Figure 1. The initial search identified 1,541 articles after duplicate removal. A total of five studies were included in the review.

### Study characteristics and risk of bias

All included studies were observational cohort studies that were published between 2014 and 2020 (Table 1). Four out of the five studies were Western (North American and/or European) and one was Asian (single-centre study from South Korea). Only one study assessed superficial gastric lesions while the remaining four investigated colonic lesions. One study used images as the method of assessment while the remaining four used recorded video clips. Gastroenterology trainees were involved in four out of the five studies.

**Table 1.** Baseline characteristics of the studies included in the systematic review.

Study author, year	Study type	Region	Lesion type	Lesions (*n*)	Method of evaluation	Participants (*n*)	GI trainees (*n*)	Experts (*n*)^a^
Aziz Aadam, 2014^18^	Obs	USA	Colonic	6	Video clips	154	34	24
Cocomazzi, 2020^19^	Obs	Italy	Colonic	25, 70^b^	Images, video clips	13	6	7
Kim, 2017^20^	Obs	South Korea	Colonic	50	Video clips	30	6	9
Ribeiro, 2018^21^	Obs	Europe	Gastric	54	Images	8	2	4
van Doorn, 2014^9^	Obs	Europe, North America	Colonic	85	Video clips	7	0	7

Obs, observational study; GI, gastroenterology; USA, United States of America.

^a^Expert defined as an endoscopist who has performed over 1,000 colonoscopies and/or performs complex polypectomy.

^b^First value represents number of lesions depicted as images; second value represents number of lesions shown in video clips.

Study quality is summarized in Table 2. Three studies (60 percent) were classified as high quality.^9,19,21^ The most common limitations found across the studies were methodologic. Only one study included a sample size calculation while none of the studies described adequate sampling methods for their selection of endoscopists. Two studies failed to report measures of statistical uncertainty for their estimates of reliability.^18,20^

**Table 2.** Study quality and risk of bias assessment adapted from GRRAS.^11^

Section	Aziz Aadam, 2014^18^	Cocomazzi, 2020^19^	Kim, 2017^20^	Ribeiro, 2018^21^	van Doorn, 2014^9^
TITLE AND ABSTRACT					
Identify in the title or abstract that interrater reliability or agreement was investigated.	N	Y	Y	Y	Y
INTRODUCTION					
Name and describe the diagnostic or measurement device of interest explicitly (Paris classification).	Y	Y	Y	Y	Y
Specify the subject population of interest.	Y	Y	Y	Y	Y
Specify the rater population of interest (if applicable).	Y	Y	Y	Y	Y
Describe what is already known about reliability and agreement and provide a rationale for the study (if applicable).	Y	Y	Y	Y	Y
METHODS					
Explain how the sample size was chosen. State the determined number of raters, subjects/objects, and replicate observations.	Y	N	N	Y	Y
Describe the sampling method.	N	N	N	N	N
Describe the measurement/rating process (e.g., time interval between repeated measurements, availability of clinical information, blinding).	Y	Y	Y	Y	Y
State whether measurements/ratings were conducted independently.	N	Y	N	N	N
Describe the statistical analysis.	Y	Y	Y	Y	Y
RESULTS					
State the actual number of raters and subjects/objects which were included and the number of replicate observations which were conducted.	Y	Y	Y	Y	Y
Describe the sample characteristics of raters and subjects (e.g., training, experience).	Y	Y	Y	Y	Y
Report estimates of reliability and agreement including measures of statistical uncertainty.	N	Y	N	Y	Y
DISCUSSION					
Discuss the practical relevance of results.	Y	Y	Y	Y	Y
TOTAL^14^	10	12	10	12	12

### Interobserver reliability of the Paris classification:

The IOR of the Paris classification ranged from moderate (*κ* = 0.42) to substantial (*κw* = 0.65), where four out of the five studies reported only moderate reliability.^9,18–20^ All four of these studies assessed colonic lesions, whereas the only study that reported a substantial IOR evaluated gastric lesions.^21^Table 3 depicts various other objective measures for IOR of the Paris classification across all studies. While all included studies presented a kappa statistic, only one was weighted.^21^ Two of the studies noted a proportion of agreement (PA) value for reliability and only one measured Gwet’s AC1 coefficient.^20^

**Table 3.** Interobserver reliability of the Paris classification across studies.

Study	Participants (*n*)	*κ* (95 percent CI)	*κw* (95 percent CI)	PA (95 percent CI)	Gwet’s AC1
Aziz Aadam, 2014^18^	154	0.48	N/R	N/R	N/R
Cocomazzi, 2020^19^	13	0.54 (0.43–0.65)	N/R	N/R	0.60 (0.50–0.70)
Kim, 2017^20^	30	0.51	N/R	N/R	N/R
Ribeiro, 2018^21^	8	N/R	0.65 (0.45–0.82)	0.91 (0.87–0.95)	N/R
van Doorn, 2014^9^	7	0.42 (0.38–0.46)	N/R	0.67	N/R

*κ*, kappa statistic; *κw*, weighted kappa statistic; PA, proportion of agreement; N/R, not reported.

A simplified morphological classification system was proposed by van Doorn et al which classified lesions into three categories: pedunculated (Ip, Isp), elevated (Is, IIa, IIb), and depressed (IIc, III), where the “elevated” category combined sessile (Is) and flat (IIa, IIb) lesions. The study authors reported that the IOR of this system was still moderate, despite demonstrating a higher reliability score of *κ* = 0.55, 95 percent CI [0.51–0.58]. Cocomazzi et al externally validated this simplified morphological system in their study by finding a similar increase in reliability, with a *κw* = 0.68, 95 percent CI [0.58–0.78]. The simplified system in Cocomazzi’s study evaluated the performance of nine pedunculated (Ip, Isp), fifty-two elevated (Is, IIa, IIb, IIa+Is), and nine depressed (IIc, IIa+IIc, Is+IIc). The breakdown of lesion subtypes was not reported in van Doorn’s study.

### Educational intervention

Three out of the five studies conducted an educational training intervention to assess the effects on the IOR of the Paris classification. While Cocomazzi et al. and Kim et al. both noted an increase in the IOR post-educational intervention, it remained in the moderate to substantial range. van Doorn et al. noted a decrease in IOR that resulted in a categorical decrease in kappa to “fair”. When stratifying by subgroup, while Cocomazzi’s educational intervention significantly increased IOR amongst experts and decreased it amongst trainees, Kim’s educational intervention increased IOR in both experts and trainees. Two out of the three studies assessed the late effects of their educational intervention on study participants at three (van Doorn et al.) and four months (Kim et al.). Kim et al. found a greater increase in IOR in the early versus late assessment intervals for both experts and trainees. However, this study observed that the experts experienced a greater decline in IOR at the four-month follow-up interval in comparison to the trainee group. A summary of these interventions and their effects on reliability are described in Table 4.

**Table 4.** Summary of educational interventions and effect on IOR of the Paris classification.

Study author, year	Description of educational intervention	Number of sessions	Lag time following intervention^a^	Pre-intervention IOR (95 percent CI)	Post-intervention IOR (95 percent CI)
Cocomazzi, 2020^19^	All participants received learning materials and attended an 1-h conference regarding the Paris classification. They then evaluated twenty-five still endoscopic images of colonic lesions retrieved from literature (pre-intervention IOR). Respondents were then made aware of the correct answers and given a chance to address mistakes in a meeting. On the same day, they subsequently evaluated seventy video clips of colonic lesions that were 10 s to 4 min in length (post-intervention IOR). Video clips were obtained from colonoscopies performed at their endoscopy unit.	One	0 months	All: *κw* = 0.54 (0.43–0.65) Experts: *κw* = 0.47 (0.34–0.60) Trainees: *κw* = 0.63 (0.50–0.77)	All: *κw* = 0.61 (0.55–0.67) Experts: *κw* = 0.61 (0.54–0.69) Trainees: *κw* = 0.59 (0.51–0.67)
Kim, 2017^20^	Participants evaluated sixty video clips of various colonic lesions that were 10–15 s in length (pre-intervention IOR). They were then presented a 20 min education program with twenty learning video clips illustrating the Paris classification. Following this, they re-evaluated the same sixty video clips in a different order (post-intervention IOR, early). This was repeated four months later (post-intervention IOR, late). Video clips were obtained from colonoscopies done by two experienced GI staff.	Two	Early = 0 months Late = 4 months	All: *κ* = 0.510 Experts: *κ* = 0.533 Trainees: *κ* = 0.514	All: Early: *κ* = 0.618 Late: *κ* = 0.58 Experts: Early: *κ* = 0.713 Late: *κ* = 0.544 Trainees: Early: *κ* = 0.631 Late: *κ* = 0.616
van Doorn, 2014^9^	Participants evaluated eighty-five video clips of various colonic lesions that were 10–25 s in length (pre-intervention IOR). Respondents were restricted to watch the clips up to three times. Three months later, they were provided a 20 min computer-based training module consisting of four steps including thirty-two learning images outlining the Paris classification^b^. Following this, they reassessed the original eighty-five video clips (post-intervention IOR). Study investigators obtained the video clips from the COCOS trial.^22^	One	3 months	Experts: *κ* = 0.42 (0.38–0.46)	Experts: *κ* = 0.38 (0.35–0.41)

IOR, interobserver reliability; GI, gastroenterology.

^a^Lag time refers to the time between educational intervention and post-intervention assessment.

^b^Step 1, introduction and basics; step 2, video and image examples of eight polyps with different morphologies (Ip, Is, IIa, IIb); step 3, training (learning set of thirty-two images); step 4, feedback on learning set.

### Other reported outcomes

Three studies reported IOR for gastroenterology trainees, all of which ranged from fair to moderate.^19–21^ When assessing the IOR of experts alone (defined as an endoscopist who has performed over 1,000 colonoscopies and/or performs complex polypectomy), Cocomazzi et al. noted a lower IOR (*κ* = 0.47, 95 percent CI [0.34–0.60]) when compared to all observers (*κ* = 0.54, 95 percent CI [0.43–0.65]) or trainees (κ=0.63, 95 percent CI [0.50–0.77]). Ribeiro et al. observed otherwise, with an increase in IOR with the experts in the assessment of gastric lesions. There was no substantial difference between trainees and experts in the study by Kim et al. Only two studies reported an IOR of lesion size.^9,18^ van Doorn et al. reported a substantial IOR (*κ* = 0.72, 95 percent CI [0.65–0.79]) when the size of the lesions was classified into three categories: diminutive (<6 mm), small (6–9 mm), or large (>9 mm). When classifying colonic lesions as polypoid and non-polypoid, van Doorn et al. reported no substantial increase in reliability as the IOR remained only moderate (*κ* = 0.43, 95 percent CI [0.38–0.49]). Both IIc gastric lesions and colonic laterally spreading tumours (LSTs) as reported by Ribeiro et al. and Cocomazzi et al., respectively, showed only a moderate IOR as well. A substantial IOR was observed by Ribeiro et al. with the addition of narrow-band imaging in the assessment of superficial gastric lesions (*κw* = 0.70, 95 percent CI [0.48–0.88]).

## Discussion

Since its development in 2002, the Paris classification became widespread in its use as a clinical decision-making tool and in comparative research to study differences in superficial gastrointestinal tract lesion morphology. Despite its endorsement by multiple professional societies,^23–25^ no validity and reproducibility studies existed until 2014. van Doorn et al. was the first group to identify a moderate IOR (*κ* = 0.42) for this classification system amongst international experts identifying colorectal neoplastic lesions.^9^ Subsequent study data—while limited—have demonstrated variable results amongst endoscopists globally.

In this study, we systematically reviewed data from eligible studies to assess IOR for the Paris classification. From 211 superficial colonic neoplasms assessed by 204 endoscopists across 4 studies, we found that the IOR of the Paris classification is consistently moderate (range, *κ* = 0.42, 95 percent CI [0.38–0.46]) to *κ* = 0.54, 95 percent CI [0.43–0.65]). The sole study in our review that demonstrated a substantial IOR evaluated gastric polyps.^21^ Given that there was only one study (with eight endoscopists) that focused on gastric lesions, further data are required to reliably conclude possible differences in IOR when comparing lesion type.

We observed a high degree of variability in the methodologic approach to the educational interventions conducted by each of the three studies that assessed this variable. While both van Doorn et al. and Cocomazzi et al. report contradicting outcomes post-educational intervention, neither of these two studies demonstrate a statistically significant change in IOR. We note that although Kim’s group reported an increase in interrater reliability following their educational intervention, their data cannot be reliably interpreted due to missing measures of statistical uncertainty. Further prospective studies with standardized programs are required to determine the impact of educational training on the IOR of the Paris classification amongst both trainees and experts at early and late follow-up intervals.

A simplified classification system was proposed by van Doorn et al. and externally validated by Cocomazzi’s group. Both groups reported a slightly improved, moderate to substantial IOR in the assessment of colonic lesion morphology. Combining Paris Is, IIa, and IIb lesions into an “elevated category”, and Paris IIc and III into a “depressed category” increases reliability amongst endoscopists given that there is known to be a high amount of variation in the proportion of lesions identified as “flat.”^9^ This simplified system is appropriate as the risk of dysplasia and submucosal invasion are similar within these categories.^26^ As indicated by Cocomazzi et al., a major drawback to this system, however, is an inability to classify LSTs, which have prognostically and therapeutically relevant features of elevation or depression (e.g., IIc + IIa).^19,27^ Additional investigation is required to address these deficiencies and to determine the ability of a simplified classification system in predicting submucosal invasion.

There are limitations to our study. First, there is significant heterogeneity and data incongruence across the included studies in the review. This is likely explained by the variability that exists in the available methods of measuring and representing reliability amongst users. While each study consistently presented IOR in the form of kappa statistics, Ribeiro et al applied a “weighted” kappa (*κw*). As this kappa allows disagreements to be weighted differently, interpreting this data in the context of non-weighted kappa studies should be cautioned. Similarly, van Doorn et al. and Kim et al. both used Fleiss kappa, a specific type of interrater reliability measure when two or more raters are involved.^15^ Second, given a low sample size and significant data variability across the five studies, we were unable to conduct meaningful statistical analyses from this review due to the risk of introducing selection bias. Furthermore, while there are some hypothesis-driven theories available in the literature, there is no validated or universally accepted method for the analysis of multiple kappa statistics from different populations.^28^

Nonetheless, this is the first study to have systematically presented important IOR data for the Paris classification. As a prominent system that is often utilized as one of the initial steps in gastrointestinal polyp characterization, this review has established an emerging platform for ongoing research to improve its reliability amongst endoscopists. Our findings suggest that the IOR of the Paris classification is only moderate for superficial colonic neoplastic lesions. While the Paris classification has been shown to predict the risk of submucosal invasion for certain subtypes of precancerous lesions, this review supports that its sole use in the initial endoscopic evaluation of superficial colonic neoplasms should be cautioned. When available, adjunctive endoscopic characterization techniques such as mucosal and vascular surface pattern examination should also be employed to further enhance the risk assessment of such lesions in guiding further management decisions. For gastric lesions, additional prospective observational studies are required to confirm whether the reliability of this classification system is higher. Finally, although educational interventions did not significantly improve IOR amongst endoscopists, further investigation with standardized training programs is warranted amongst both experts and trainees.

## Supplementary Material

gwad039_suppl_Supplementary_Tables

## Data Availability

The authors confirm that the data supporting the findings of this study are available within the article and its supplementary materials.

## Protocol registration number

Prospero: CRD42021247175
